# Improving Sound Absorption Properties Using 3D-Printed ASA Concentric Tubular Structures with Intermediate Lattice Inserts

**DOI:** 10.3390/polym18101193

**Published:** 2026-05-13

**Authors:** Martin Vasina, Katarina Monkova, Adrian Vodilka

**Affiliations:** 1 Faculty of Technology, Tomas Bata University in Zlin, Vavreckova 5669, 760 01 Zlin, Czech Republic; 2 Faculty of Mechanical Engineering, VSB - Technical University of Ostrava, 17. listopadu 2172/15, 708 00 Ostrava-Poruba, Czech Republic; 3 Faculty of Manufacturing Technologies with a Seat in Presov, Technical University of Kosice, Sturova 31, 080 01 Presov, Slovakia; adrian.vodilka@tuke.sk

**Keywords:** 3D printing, sound absorption, concentric tubes, lattice structure, strut diameter, excitation frequency, back air cavity

## Abstract

Noise is an environmental factor that negatively affects the health of living organisms and must therefore be mitigated. One effective approach to noise reduction is the use of passive materials for sound absorption. Moreover, with the increasing use of 3D printing technology, it is now possible to produce complex material structures for noise reduction that cannot be manufactured using conventional manufacturing techniques. This study investigates the sound absorption performance of novel 3D-printed concentric tubular structures made of acrylonitrile styrene acrylate (ASA) with intermediate lattice inserts. The sound absorption properties of these structures were experimentally evaluated in the frequency range of 200–1600 Hz using a two-microphone acoustic impedance tube. Various factors influencing sound absorption properties were investigated, including the number of concentric tubes, sample height, strut diameter, and back air cavity thickness. The experimental results show that the sound absorption performance depends significantly on the design parameters of the proposed system. The average sound absorption coefficient (*α_avg_*) increased with the number of concentric tubes and reached a maximum value of 0.264 for the configuration with five tubes. The highest sound absorption peak (*α_max_* = 0.623) was achieved for the structure with two concentric tubes, a strut diameter of 3 mm, a height of 30 mm, and a back air cavity of 10 mm at a frequency of approximately 1548 Hz. Furthermore, increasing the strut diameter and sample height generally improved sound absorption performance, while the presence of a back air cavity significantly shifted the absorption peak toward lower frequencies, thereby enhancing low-frequency sound absorption.

## 1. Introduction

Noise is a significant environmental factor that poses a considerable risk to human health. Long-term exposure to elevated noise levels can result in irreversible hearing damage and hearing loss. In addition, chronic exposure to noise is associated with sleep disturbances, fatigue, stress, anxiety, and reduced cognitive performance [1]. Noise control methods are generally classified into active and passive approaches. Passive noise control (PNC) reduces noise through vibration damping, sound absorption, and sound insulation using suitable materials, whereby acoustic energy is converted into other forms of energy [2]. PNC systems mainly employ porous and resonant sound-absorbing materials [3]. Porous materials, such as foams and fibrous media, dissipate sound energy through viscous and thermal losses within their pore structures, but exhibit poor low-frequency absorption and require increased thickness and weight. Resonant absorbers, including Helmholtz resonators and membrane-based systems, are effective at low frequencies. However, their narrow absorption bandwidth significantly limits their practical applications [3,4]. Active noise control (ANC) reduces noise by generating an anti-noise signal with equal amplitude and opposite phase to the unwanted environmental sound [5].

The popularity of 3D printing technology, which fabricates components layer by layer from digital models, is rapidly increasing worldwide. It is widely applied in energy, agriculture, healthcare, engineering, and other industries and can process a broad range of materials, including polymers, metals, ceramics, resins, and biomaterials [6,7]. 3D printing enables the fabrication of complex geometries that are difficult to achieve using conventional techniques, while also reducing material waste and energy consumption [8]. At present, 3D printing technology is widely used to develop novel porous structures for sound absorption, ranging from simple to highly complex geometries. The acoustic performance of 3D-printed porous materials is governed by various parameters such as porosity, density, pore size and morphology, structural complexity, material composition, wall and sample thickness, incident sound frequency, and internal airflow characteristics.

The sound absorption properties of 3D-printed porous structures have been investigated in several studies. In [9], acrylonitrile butadiene styrene (ABS) and polylactic acid (PLA) specimens with square and hexagonal cell geometries and infill ratios of 10–50% were examined, showing that higher infill ratios generally improved sound absorption, although the overall sound absorption remained low. PLA specimens and hexagonal geometries exhibited superior acoustic performance compared to ABS and square cells. Similarly, Bocanegra et al. [10] reported low absorption coefficients (*α_max_* ≈ 0.2) for thin perforated 3D-printed panels, with elliptical perforations outperforming circular ones. In addition, Pop et al. [11] demonstrated that PLA panels with cubic cell geometries achieved maximum sound absorption at infill densities of 60–70%, depending on the panel type. King et al. [12] investigated thin micro-perforated panels (MPPs) with circular holes and found that the peak absorption frequency (*f_p_*) decreased with increasing hole spacing, hole size (perforation ratio), and air cavity depth. Similarly, for 3D-printed porous samples with a square structure, *f_p_* decreased with increasing thickness and perforation ratio. However, for both types of these structures, enhanced sound absorption was observed only within a narrow band around *f_p_*. Akiwate et al. [13] studied 3D-printed perforated panels with circular and non-circular (i.e., square and triangular) perforations backed by a honeycomb structure. For circular perforations, sound absorption increased with decreasing aperture ratio. Panels with triangular perforations showed the highest absorption and lowest peak frequency *f_p_*, while the opposite trend was observed for circular perforations. Additionally, *f_p_* decreased with increasing back hexagonal cell length.

Monkova et al. [14] investigated 3D-printed ABS materials with various structural geometries, including Cartesian, Octagonal, Rhomboid, and Starlit patterns. The Starlit structure showed superior sound absorption due to its more complex pore geometry. Sound absorption increased with higher sample volume ratio, material thickness, and air gap size, particularly at lower frequencies. Similar trends were observed for 3D-printed open-porous polyamide hexagonal prism lattices, as confirmed by numerical simulations of specific airflow resistance [15]. Samples with a 2 mm outer shell exhibited better sound absorption compared to fully lattice-structured samples, while the lattice cell rotation angle had a negligible effect. The influence of perforation cross-sectional variation on the sound absorption of 3D-printed PLA MPPs was investigated in [16]. Samples with an 8 mm perforation diameter showed significantly lower sound absorption than those with a constant 1 mm diameter. MPPs with varying cross-sections (convergent–divergent, divergent–convergent, convergent, and divergent) exhibited improved sound absorption over a wider frequency range compared to the constant 1 mm perforation. Zielinski et al. [17] investigated the sound absorption of 3D-printed periodic cellular structures fabricated using stereolithography (SLA) and ColorJet Printing (CJP) technologies. SLA samples, printed from photopolymer resin, had single porosity, whereas gypsum-based CJP samples were either impregnated with cyanoacrylate to eliminate micropores or left non-impregnated, retaining a double-porosity structure. The highest absorption was observed in double-porosity CJP samples, while SLA samples exhibited slightly lower absorption than the impregnated CJP samples. Sound-absorbing 3D-printed fibrous structures, produced using fiber bridging and extrude-and-pull methods, were investigated in [18]. Results showed that bridging samples, with consistently thicker fibers, higher fiber density, and smaller effective pore sizes, exhibited enhanced sound absorption compared to extrude-and-pull samples, indicating that acoustic behaviour can be effectively tuned by adjusting printing parameters.

Enhanced sound absorption properties can be achieved in material structures with complex geometries. Shen et al. [19] compared the sound absorption properties of 3D-printed PLA samples with standard infill patterns (aligned rectilinear, honeycomb, triangles, gyroid, and grid) and Kresling origami (KO) structures. The results showed that more complex geometries (especially gyroid and KO patterns) exhibited significantly enhanced acoustic absorption performance compared to conventional infill (i.e., rectilinear, triangular, and grid) patterns. The sound absorption properties of multisheet gyroid lattice structures fabricated using SLA were investigated in [20], revealing that two-sheet gyroid structures exhibited significantly better broadband sound absorption than one-sheet configurations. In [21], sound absorption was studied in uniform and layered gyroid and diamond triply periodic minimal surface (TPMS) porous absorbers. It was found that higher relative density increases the airflow resistivity of the absorbers, thereby enhancing their absorption performance. Diamond geometries in uniform absorbers exhibited superior sound absorption compared to gyroid samples with the same relative density. Furthermore, the stacking sequence (series or parallel) and the gradient orientation relative to the incident wave direction had a strong influence on the sound absorption behaviour. Chouhan et al. [22] investigated the sound absorption of four TPMS lattice structures fabricated using SLA technology. They reported that gyroid and diamond lattices exhibited superior sound absorption compared to lidinoid and split P lattices. Additionally, it was observed that the sound absorption performance increased with decreasing lattice porosity and increasing sample thickness. The acoustic properties of a metamaterial consisting of cylindrical holes of varying depths arranged circumferentially and divided into different cavities by spacers were experimentally and numerically studied in [23]. It was found that the experimental and simulated frequency dependencies of the sound absorption coefficient (SAC) were in good agreement, with an average SAC of 0.87 observed in the frequency range of 490–1100 Hz. 3D-printed PLA-based MPPs manufactured using three nozzle diameters, three types of polylactic acid filaments, and six internal configurations incorporating labyrinthine zigzag channels were investigated in [24], achieving a maximum SAC of 0.93 at a frequency of 500 Hz under optimal conditions.

Sound absorption properties of structured materials can also be tailored using tunable acoustic absorbers. High sound absorption (average SAC > 0.92) in the frequency range of 500–1150 Hz was achieved using a tunable acoustic absorber composed of five metamaterial cells, each containing nine parallel-connected Helmholtz resonators with adjustable apertures [25]. Chen et al. [26] proposed a subwavelength aperiodic low-frequency sound absorber consisting of three parallel split-ring units, achieving high sound absorption (*α* > 0.9) in a broadband frequency range between 229 and 457 Hz. Furthermore, an extended aperiodic configuration consisting of nine coupled units provided improved broadband performance in the range of 272–394 Hz. A double-layer honeycomb microperforated plate structure with screw-adjustable cavity depth was developed by Yan et al. [27] to enable tunable and broadband sound absorption. The results showed that geometric parameters such as pore diameter, plate thickness, and perforation rate affect absorption efficiency, while mechanical adjustment of the cavity depth shifts the absorption peak and frequency range. Xu et al. [28] proposed a tunable absorber consisting of three layers of annular micro-slit tubes. By rotating the central micro-slotted tube between 0° and 180°, continuous tuning was achieved in the operating frequency range from 280 to 572 Hz, exhibiting high sound absorption (*α* > 0.9). Zhang et al. [29] studied tunable mode-switchable metamaterials based on annular micro-perforated panel structures, which enable switching between serial and parallel cavity configurations, thereby achieving both enhanced low-frequency absorption and broadband acoustic performance. Furthermore, the introduction of a correction factor accounting for the effective sound propagation length improves the accuracy of classical impedance models when applied to complex cavity geometries. As a result, efficient sound absorption has been achieved in the frequency range of 270–960 Hz with a subwavelength thickness of only 29 mm, highlighting the strong potential of such designs for compact and high-performance noise control applications. An acoustic metastructure, in which porous materials (polyester, glass wool, and ceramic fibers) are enclosed within a perforated front panel and segmented cavities, thereby increasing low-frequency sound absorption while maintaining ventilation, was investigated in [30]. By adjusting the length and type of porous material, the system achieved high sound absorption (α > 0.8) in the low-frequency range of approximately 400–700 Hz. Compared to bulk porous materials, which exhibit poor low-frequency performance without substantial thickness, the metastructure enables strong absorption using significantly thinner porous inserts.

Roncen [31] investigated the nonlinear impedance of acoustic liners under complex source excitations using experiments and the Impulse Response Time-Domain Impedance Boundary Condition (IR-TDIBC) model. The results show that the impedance strongly depends on the spectral content, amplitude, and phase relationships of multitone and broadband signals, with instantaneous particle velocity identified as the key governing parameter. The IR-TDIBC model accurately reproduces this nonlinear behaviour across various excitation conditions. Furthermore, flow-induced noise increases acoustic resistance while affecting reactance in a frequency-dependent manner. These findings highlight the importance of accounting for both complex source excitations and flow-induced noise in impedance modelling of acoustic liners, thereby improving prediction accuracy for practical aeroacoustic applications.

Beyond the structures discussed above, a wide range of additively manufactured material structures for sound absorption has been reported, with their designs and acoustic properties reviewed in detail in [32,33,34,35]. This study focuses on the sound absorption properties of novel 3D-printed ASA concentric tubular structures with intermediate lattice inserts. Specifically, it examines the influence of the number of concentric tubes, the sample height, the strut diameter, the back air cavity thickness within the acoustic impedance tube, and the excitation frequency of acoustic waves on the sound absorption performance of 3D-printed polymer materials produced using the Fused Filament Fabrication (FFF) technique. To the best of the author’s knowledge, only a limited number of studies have addressed structures combining concentric tubular geometries with internal lattice inserts. However, their systematic acoustic characterization and parametric evaluation have not yet been sufficiently explored.

Although a wide range of 3D-printed porous and metamaterial absorbers has been described, most studies focus either on conventional lattice structures with relatively simple flow paths or on highly optimized metamaterial designs that achieve high absorption coefficients at the expense of structural complexity and manufacturability. In contrast, the combined effect of macroscopic guiding elements (e.g., concentric tubes) and internal lattice inserts has not been systematically investigated. From this perspective, the proposed design represents a transitional configuration between purely lattice structures and multi-cavity resonant systems, utilizing the combined effect of flow channel restriction and internal dissipation. Therefore, the main contribution of this work lies in the systematic investigation of this hybrid concept, with emphasis on manufacturability, structural stability, and tunable acoustic response, rather than maximizing peak sound absorption values.

This study should therefore be regarded more as a proof-of-concept and a parametric investigation than an attempt to create a new theoretical framework for sound absorption. In this context, the relationship between structure and acoustics in the proposed system arises from the interaction between channel-guided wave propagation and lattice-induced viscous–thermal dissipation. The observed trends can be interpreted in terms of changes in the effective airflow resistance, tortuosity, and acoustic path length caused by changes in geometric parameters.

## 2. Materials and Methods

### 2.1. Sample Design

**Figure 1:**
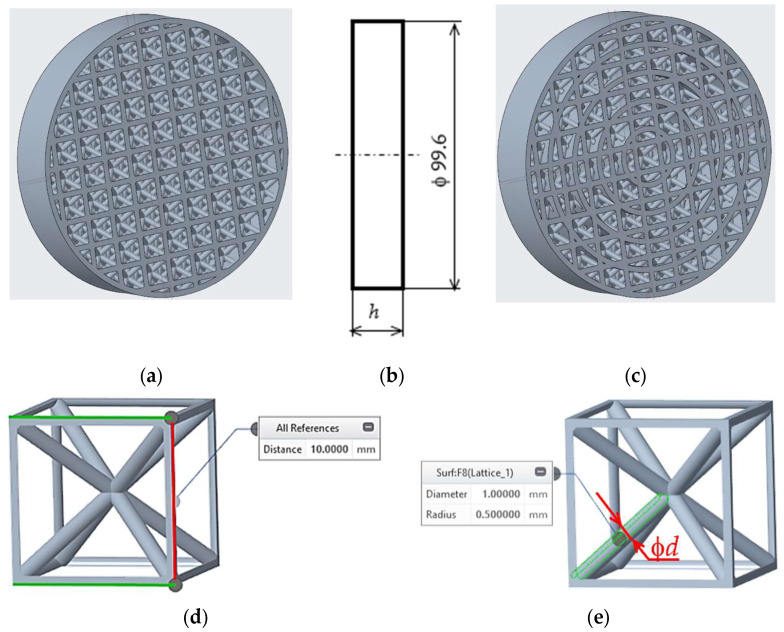
Sample design, (**a**) 3D model with one outer tube (*T* = 1); (**b**) basic dimension with the height *h* as a varied testing parameter; (**c**) 3D model of a sample model with 5 concentric tubes (*T* = 5); (**d**) a basic BCC cell showing sizes of 10 × 10 × 10 mm designed for internal structure of a cylindrical sample; (**e**) a cell with a strut diameter d indication (*d* = 1 mm).

Virtual 3D models of the samples were generated using PTC Creo 11 software (PTC Inc., Boston, MA, SUA) with a count of *T* = 1, 2, 3, and 5 concentric tubes with a constant wall thickness of 2 mm and evenly distributed on the diameter from the centre of the sample according to the number of tubes used (see Figure 1a,c). The sample’s outer diameter of 99.6 mm was generated with the intention of the final samples being inserted into the sound testing device and thus had to be dimensionally accurate to ensure the absence of any gap between the testing apparatus and the samples under evaluation, while the height *h* of the specimens (Figure 1b) varied in sizes 10, 20, and 30 mm.

The samples were filled with an internal intermediate structure with a BCC (Body-Centred Cubic) cell dimensions of 10 × 10 × 10 mm (Figure 1d) distributed uniformly in the perpendicular *x*, *y*, *z* directions to fill in the body of the sample. The strut diameter *d* (Figure 1e) within the BCC cell was set to values of 2, 2.5, and 3 mm based on manufacturability and meaningfulness of testing the samples.

A total of 36 virtual models (3 heights (*h*) × 3 strut diameters (*d*) × 4 concentric tubes (*T*)) were generated using the software and exported to the *stl format readable by the software of the 3D printing machine.

### 2.2. Sample Material

The material chosen for the research was ASA (Acrylonitrile Styrene Acrylate), specifically PREMIUM LINE ASA in natural colour from C-TECH (C DISTRIBUTION s.r.o., Brno, Czech Republic). It is a top-quality filament made for demanding professional and industrial uses. Its impressive UV stability and weather resistance make it suitable for both indoor and outdoor projects—even under tough conditions. Because it is formulated without plasticizers, Premium ASA boasts strong mechanical features like high rigidity, excellent strength, and superior impact resistance. The physical properties of ASA material are listed in Table 1.

ASA stands out in particular for its higher dimensional stability during printing, lower tendency to twist, and better integrity of the interface layers in complex geometries, such as inclined beams in BCC lattice structures, compared to more common polymers such as PLA or ABS. This allows for a more accurate reproduction of geometry, which is crucial in experiments where the influence of geometric parameters on the acoustic response is investigated, since the accuracy of the geometry is essential for ensuring comparable results between individual samples. At the same time, ASA has the advantage over conventional ABS in lower thermal expansion and better adhesion of interlayers, while maintaining similar toughness and impact strength. Unlike PLA, which allows for easy printing and a smoother surface but exhibits higher brittleness and lower thermal resistance, ASA provides greater long-term stability of samples and less susceptibility to degradation during handling or repeated acoustic measurements.

In terms of acoustic properties, the ASA material itself has similar density and elastic modulus characteristics in the low and mid-frequency range to ABS, which means that the differences between the two materials are likely to be more evident in secondary effects, such as internal attenuation and interaction with the geometry of the porous structure. ASA has a slightly lower internal loss factor than PLA, which may lead to a smaller material component of attenuation, i.e., the effect of the pore morphology itself and the topology of the lattice structures is more pronounced. This is desirable from the point of view of investigating acoustic trends, as it allows for better isolation of the effect of geometry from material attenuation. This suggests that the effect of the implemented non-porous barriers on the resulting absorption can be more clearly interpreted as a geometric effect, without significant masking by material behaviour. On the other hand, ASA exhibits slightly higher surface hardness and lower porosity between individual fibers compared to PLA or PETG, which may lead to lower microporosity in the walls of the beams. This factor may reduce the degree of microscopic viscous-thermal interaction with air, which could lead to ASA demonstrating lower overall absorption compared to materials with higher microporosity. However, in a comparative study, this effect can be viewed positively, as it again contributes to the fact that the measured differences in absorption are more likely to be a consequence of the used topology and the introduced barriers than the inconsistencies of the material structure. ASA thus represents a suitable compromise between mechanical stability, sufficient stiffness and relatively neutral acoustic behaviour.

The material’s sturdy building ensures stable dimensions and lasting performance, making it perfect for parts that need to withstand stress or harsh outdoor environments. In the case of ABS, the risk of deformation and surface irregularities due to cooling stress is significantly higher, while PLA could suffer from deformations related to thermal softness and creep effect in larger samples. 3D prints made with Premium ASA remain durable over time, keeping their shape and color even after exposure to sunlight, moisture, and changing temperatures. This reliability makes Premium ASA ideal for automotive components, drone body parts, electrical enclosures, outdoor fixtures, tools, prototypes, and products designed for regular use. All these factors supported the choice of ASA as a suitable material for systematic acoustic experiments, the aim of which was to reveal the relationships between geometry and acoustic response, rather than to optimize a specific case of sound transmission.

### 2.3. Production of 3D-Printed Polymer Samples

The Fused Filament Fabrication (FFF) technique was employed to produce the testing samples, utilizing the Anycubic Kobra S1 Combo 3D printer (Anycubic, Shenzhen, China). This desktop device adopts CoreXY architecture, which provides stable movement for the machine’s mechanical components. Notably, Kobra S1 features an enclosed print chamber, a design consideration demonstrated to improve process stability by minimizing the impact of external environmental fluctuations—most significantly, ambient air temperature, which has a substantial effect on model quality. The print chamber is maintained at a consistent temperature between 50 °C and 60 °C, primarily through heat generated by the printing pad, ensuring optimal conditions for fabricating parts from materials intended for demanding applications. The Anycubic Slicer Next version 1.3.9.3 (Anycubic, Shenzhen, China) was chosen as the toolpath generator. It facilitates the incorporation of material shrinkage compensation following the measurement of the actual dimensions against their nominal equivalents.

The production parameter settings were selected based on the authors’ previous experience with 3D printing of cellular materials [38,39,40,41], which were modified to meet the needs of the manufactured lattice structure with respect to the ASA material. The need to achieve an outer diameter of 99.6 mm was assumed to ensure an accurate fit with the inner diameter of the sound test tube.

The native diameter of the 3D model in the preliminary samples was set to 100 mm (due to easier computation in dimension changing during the next steps). After the first samples manufacture, the real diameter was measured to be 99.4 mm due to material shrinkage. Given this reference value, the data for the final samples were recalculated, while the maximum fan speed was reduced from the original 80% to 10%, given that the initial setting of 80% resulted in cooling of the entire 3D printed model during the manufacturing process, although the goal was to cool only the material extrusion area. This caused an issue with uncontrolled material shrinkage and the creation of a warping effect, which caused production failures in initial tests. Subsequently, the fan speed was kept at 10% due to the presence of overhangs (oblique struts) in the 3D model structure.

**Figure 2:**
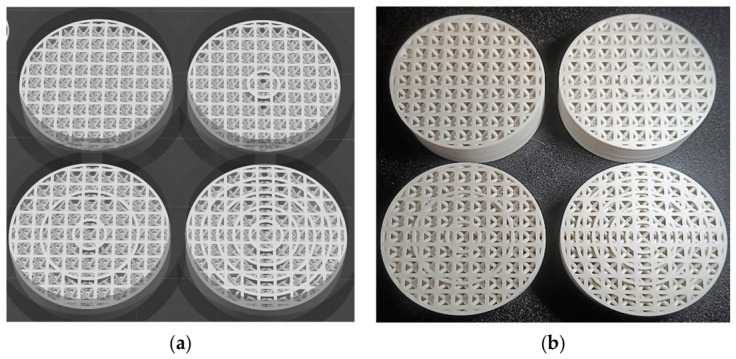
The print task of physical models. (**a**) Display of the print task in Anycubic Slicer Next. (**b**) Display of the print task after completion.

To facilitate the gradual cooling of the layers and thus mitigate the effect of warping, the 3D printed models were distributed across the print platform, with four physical models printed in a single print job. This extended the printing time for each layer, allowing for a more gradual cooling process and the release of internal stress. The configuration of the printing bed, together with the three-dimensional models of the print task, is illustrated in Figure 2a, while an image of the completed print task can be seen in Figure 2b.

**Figure 3:**
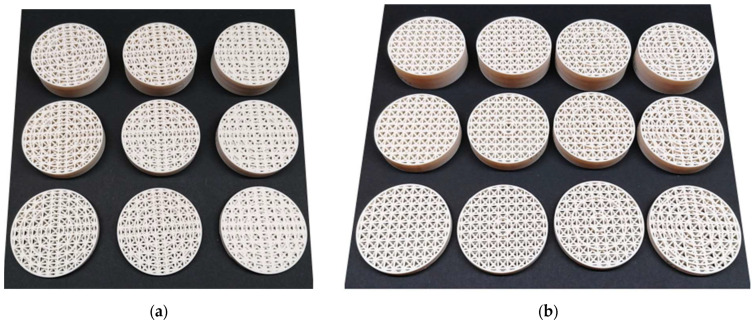
Example of manufactured samples, (**a**) series of samples with the same number of *T* = 5 concentric tubes, but with different strut diameters at different heights; (**b**) series of samples with the same strut diameter *d* = 2 mm, but with different numbers of concentric tubes at different heights *h* = 10, 20, and 30 mm.

After several attempts to produce preliminary samples and correct the input factor settings to ensure sample quality and sizes, the final parameters were set as shown in Table 2, while examples of samples from two different views of the series are presented in Figure 3.

### 2.4. Measurement Methodology

The ability of a material to dampen sound is expressed by the sound absorption coefficient *α*, which is given by Equation (1) [42]:

(1) $$\alpha =\frac{E_{A}}{E_{I}}=1-\frac{E_{R}}{E_{I}}=\frac{E_{T}+E_{D}}{E_{I}}$$

where *E_A_* is the absorbed sound energy, *E_I_* is the incident sound energy, *E_R_* is the reflected sound energy, *E_T_* is the transmitted sound energy, and *E_D_* is the dissipated energy. The sound absorption coefficient ranges from 0 to 1, where a value of 1 indicates perfect absorption of sound energy (no reflection), and a value of 0 indicates that the material does not absorb any incident sound energy but instead reflects it completely. It is influenced by several factors, including the excitation frequency of incident acoustic waves, material type, structure, density, thickness, and temperature [43].

Frequency dependencies of the normal incidence sound absorption coefficient α for the investigated 3D-printed polymer samples were experimentally determined using a two-microphone acoustic impedance tube (BK 4206) in combination with a power amplifier (BK 2706) and a PULSE multi-analyzer (BK 3560-B-030) over the frequency range of 200–1600 Hz at an ambient temperature of 23 °C. All measurement equipment used in this study (BK 4206, BK 2706, and BK 3560-B-030) was manufactured by Brüel & Kjær, Nærum, Denmark. The measurements were performed using the two-microphone transfer function method in accordance with the ISO 10534-2 [44] standard, which is based on the partial standing wave principle. According to this method, the normal incidence sound absorption coefficient is expressed by Equation (2) [45]:

(2) $$\alpha =1-{\left|r\right|}^{2}=1-{\left|\frac{H_{12}-H_{I}}{H_{R}-H_{12}}\cdot e^{2jkx_{1}}\right|}^{2}$$

where *r* is the reflection factor, *H*_12_ is the pressure transfer function, *H_I_* is the transfer function of the incident acoustic wave, *H_R_* is the transfer function of the reflected acoustic wave, *j* is the imaginary unit, *k* is the wave number, and *x*_1_ is the distance between the sample surface and the microphone M_1_ positioned closer to the sample. The transfer functions are given by Equations (3)–(5):

(3) $$H_{12}=\frac{p_{2}}{p_{1}}=\frac{e^{jkx_{2}}+r\cdot e^{-jkx_{2}}}{e^{jkx_{1}}+r\cdot e^{-jkx_{1}}}$$

(4) $$H_{I}=e^{-k\cdot \left(x_{1}-x_{2}\right)j}$$

(5) $$H_{R}=e^{k\cdot \left(x_{1}-x_{2}\right)j}$$

where *x*_2_ is the distance between the sample surface and the microphone M_2_ located further away from the sample, and *p*_1_ and *p*_2_ are the complex acoustic pressures measured by the microphones M_1_ and M_2_.

As already mentioned, the sound absorption coefficient depends, among other factors, on the frequency of acoustic waves. Therefore, to compare the sound absorption properties of the tested 3D-printed materials, the average sound absorption coefficient *α_avg_* was determined using Equation (6):

(6) $$\alpha_{avg}=\frac{1}{n}\cdot \sum_{i=1}^{n}\alpha_{i}$$

where *α_i_* is the absorption coefficient at the *i*th frequency within the measured frequency range of 200–1600 Hz, and *n* is the number of frequencies at which the corresponding *α_i_* values were measured. The normal incidence sound absorption coefficient of the tested samples was also determined for different back air cavity thicknesses *a* (ranging from 0 to 100 mm) behind the investigated 3D-printed ASA samples inside the acoustic impedance tube.

Prior to the experimental determination of the frequency-dependent sound absorption coefficient, the microphones M_1_ and M_2_ were calibrated according to the manufacturer’s recommended procedure, and phase correction was performed to ensure accurate measurement of the acoustic response. To assess measurement repeatability, selected samples were measured repeatedly under identical experimental conditions. The observed variations in the sound absorption coefficient were small across the whole investigated frequency range and did not affect the overall trends of the results. All measurements were performed under controlled laboratory conditions at an ambient temperature of 23 °C, ensuring stable environmental parameters. The measurement uncertainty is considered low due to the use of a standardized two-microphone transfer function method in accordance with ISO 10534-2, which provides high repeatability for normal-incidence sound absorption measurements.

## 3. Results and Discussion

This section discusses the various factors influencing the sound absorption properties of the investigated ASA concentric tubular structures with intermediate lattice inserts.

### 3.1. Influence of Number of Concentric Tubes

**Figure 4:**
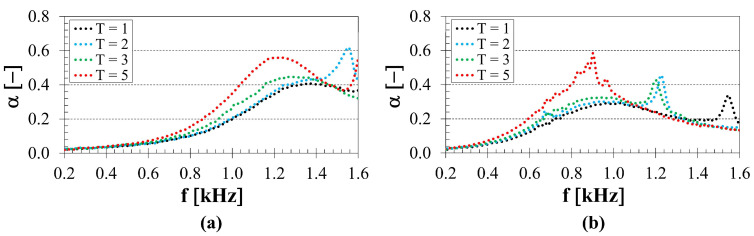
Influence of number of concentric tubes *T* on the frequency dependencies of the sound absorption coefficient; (**a**) strut diameter *d* = 3 mm, sample height *h* = 30 mm, back air cavity *a* = 10 mm, (**b**) strut diameter *d* = 3 mm, sample height *h* = 20 mm, back air cavity *a* = 40 mm.

As shown in Figure 4, the number of longitudinally inserted concentric tubes significantly influenced the sound absorption properties of the tested samples. The influence of the number of concentric tubes on the sound absorption properties of the 3D-printed ASA samples manufactured with a strut diameter of 3 mm, a height of 30 mm and a back air cavity thickness of 10 mm inside the acoustic impedance tube is demonstrated in Figure 4a. It is evident that a higher ability to absorb sound with the increasing number of concentric tubes (*T*) was observed, particularly in the mid-frequency range, namely from 600 to 1400 Hz. It was found that the average sound absorption coefficient (*α_avg_*), calculated according to Equation (6) over the entire measured frequency range (i.e., from 0.2 to 1.6 kHz), decreased with decreasing number of concentric tubes. Specifically (see Figure 4a), the coefficient *α_avg_* decreased from 0.264 (*T* = 5) to 0.217 (*T* = 3), followed by a slight further decrease to 0.215 (*T* = 2), and reached a minimum value of 0.192 (*T* = 1). A similar trend in sound absorption performance as a function of the number of concentric tubes (see Figure 4b) was also observed for the samples manufactured with a strut diameter of 3 mm, a height of 20 mm, and a 40 mm back air cavity behind the samples within the impedance tube. In this case, the coefficient *α_avg_* decreased from 0.224 (*T* = 5) to 0.199 (*T* = 3), followed by a further decrease to 0.191 (*T* = 2), and reached a minimum value of 0.183 (*T* = 1). The decrease in *α_avg_* with decreasing number of concentric tubes was also significant for the other tested samples. Only for the samples mounted directly on the solid wall without a back air cavity thickness (*a* = 0 mm) within the impedance tube, the average sound absorption properties of the tested samples remained very similar. Therefore, the influence of *α_avg_* on the number of concentric tubes was negligible in these cases, particularly for specimens manufactured with smaller heights and strut diameters.

As shown in Figure 4, the sound absorption performance generally improves with an increasing number of concentric tubes, as reflected by higher average sound absorption coefficients. This trend suggests that more complex material structures enhance the conversion of acoustic energy into heat during the propagation of acoustic waves through the material. A similar behaviour was reported in [15], where hexagonal lattice structures with an outer shell exhibited higher specific airflow resistance [46] and improved sound absorption compared to purely hexagonal lattice structures. This phenomenon can be attributed to a reduction in the effective flow cross-section caused by the additional concentric tubes, which increases the specific flow resistance and promotes mechanisms of viscous and thermal losses during the propagation of acoustic waves. As a result, a larger part of the incident acoustic energy is converted into heat. This explanation is consistent with the fundamental theory of porous sound-absorbing materials, according to which the main mechanisms of sound attenuation are viscous and thermal losses in interconnected pores [47].

In general, the ability of materials to absorb sound increases with airflow resistance up to an optimal level, beyond which excessive resistance leads to increased reflection of acoustic waves and reduced sound transmission through the material structure [48,49]. This behaviour is consistent with previously reported trends in additively manufactured porous and lattice materials, where similar relationships between structural complexity, reduced effective pore size, and enhanced acoustic dissipation have also been reported. For example, it has been demonstrated that increasing the relative density or reducing the porosity increases airflow resistance and improves the sound absorption properties of lattice- and TPMS-based structures [21,22]. Similarly, smaller pore sizes and higher tortuosity enhance viscous–thermal dissipation due to stronger interactions between the acoustic wave and the material’s internal surfaces [18,34].

Therefore, the increase in sound absorption with the number of concentric tubes can be explained by a combined effect of increased airflow resistance, a reduction in the effective flow channels, and increased dissipation of viscous and thermal energy within the complex tubular lattice structure.

### 3.2. Influence of Strut Diameter

**Figure 5:**
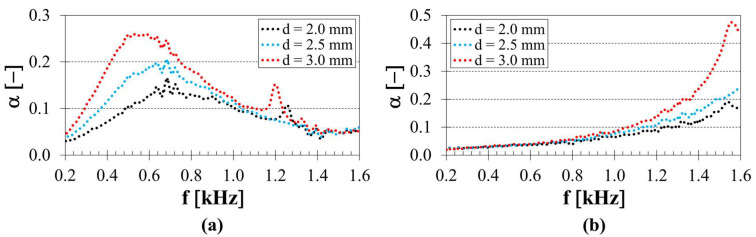
Influence of strut diameter *d* on the frequency dependencies of the sound absorption coefficient; (**a**) number of tubes *T* = 3, sample height *h* = 20 mm, back air cavity *a* = 100 mm, (**b**) number of tubes *T* = 5, sample height *h* = 30 mm, back air cavity *a* = 0 mm.

The strut diameter *d* of the 3D-printed lattice structures is a significant parameter influencing their total volumetric porosity and infill density, and consequently, their sound absorption performance. The influence of the strut diameter on the sound absorption properties of the 3D-printed ASA samples manufactured with three concentric tubes, a height of 20 mm, and a back air cavity thickness of 100 mm inside the acoustic impedance tube is presented in Figure 5a. It is evident that the samples’ ability to absorb sound increased with increasing strut diameter across a broad frequency range, namely from 0.2 to 1.2 kHz. For this reason, the average sound absorption coefficient *α_avg_* decreased from 0.137 (*d* = 3.0 mm) to 0.107 (*d* = 2.5 mm), and subsequently reached a minimum of 0.087 (*d* = 2.0 mm). Similarly, the coefficient *α_avg_* decreased from 0.121 (*d* = 3.0 mm) to 0.086 (*d* = 2.5 mm), and subsequently reached a minimum of 0.072 (*d* = 2.0 mm) for the specimens manufactured with five concentric tubes, a height of 30 mm, and without a back air cavity thickness (*a* = 0 mm) inside the acoustic impedance tube, as shown in Figure 5b. A similar increase in sound absorption with increasing strut diameter was also observed for all other samples.

The results indicate that increasing the strut diameter generally improves the sound absorption performance of the investigated lattice structures. This behaviour can be explained by the fact that, unlike a change in the number of concentric tubes (Figure 4), which primarily affects the overall topology and the number of available propagation paths, the strut diameter directly controls the local flow narrowing in individual channels. Larger strut diameters reduce the effective hydraulic diameter of interconnected pores and modify the internal airflow distribution, resulting in higher airflow resistance and stronger interaction between the acoustic wave and the solid framework. Consequently, viscous and thermal effects become more pronounced, increasing the dissipation of acoustic energy. A similar relationship between reduced pore size, increased airflow resistivity, and improved sound absorption has been widely reported for additively manufactured lattice and porous structures [21,22,34,50].

### 3.3. Influence of Sample Height

**Figure 6:**
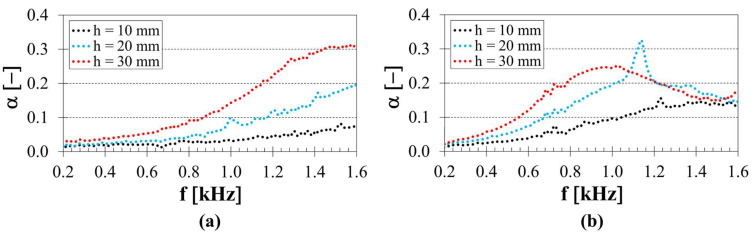
Influence of sample height *h* on the frequency dependencies of the sound absorption coefficient; (**a**) number of tubes *T* = 2, strut diameter *d* = 2.5 mm, back air cavity *a* = 10 mm; (**b**) number of tubes *T* = 5, strut diameter *d* = 2.0 mm, back air cavity *a* = 40 mm.

The height (or thickness) h of the investigated 3D-printed open-porous ASA samples is also an important factor influencing their sound absorption performance. The effect of the sample height on the frequency dependencies of the sound absorption coefficient of the 3D-printed ASA samples fabricated with two concentric tubes, a strut diameter of 2.5 mm, and a back air cavity thickness of 10 mm inside the acoustic impedance tube is shown in Figure 6a. It is evident that a greater sample height generally led to improved sound absorption properties, resulting in a decrease in the *α_avg_* coefficient from 0.145 (*h* = 30 mm) to 0.077 (*h* = 20 mm), and subsequently to a minimum of 0.036 (*h* = 10 mm). Similarly, the coefficient *α_avg_* decreased from 0.157 (*h* = 30 mm) to 0.136 (*h* = 20 mm), and subsequently to a minimum of 0.083 (*h* = 10 mm) for the samples manufactured with five concentric tubes, a strut diameter of 2.0 mm, and a 40 mm back air cavity behind the samples within the impedance tube, as depicted in Figure 6b. The increase in *α_avg_* with increasing sample height was also observed for the other samples tested.

This behaviour follows the same general trend observed for the strut diameter (Figure 5), where changes in local geometry led to changes in airflow resistivity and viscous–thermal dissipation. However, while the strut diameter primarily affects local narrowing at the pore level and the redistribution of airflow within individual channels, the sample height affects the total length of the acoustic wave path through the structure. In this regard, increasing thickness prolongs the interaction time between the incident acoustic wave and the internal rigid framework, leading to a gradual accumulation of viscous and thermal losses along the propagation direction. A similar distinction between local geometric effects and global wave propagation effects has been observed in additively manufactured lattice materials, where pore-level properties (e.g., strut size, pore aperture) significantly affect airflow resistance, while sample thickness influences low-frequency attenuation and overall energy dissipation [18,21,34,51].

Although a thicker sample improves sound absorption properties, it necessarily leads to longer printing times and higher material consumption, which increases the overall production costs of 3D-printed samples [52]. From a practical point of view, this represents a compromise between acoustic performance and manufacturability, particularly in cases where mass production is required or where cost is a critical factor. Therefore, simply increasing the thickness of the structure may not be the most effective solution for noise reduction in practical applications. In such cases, improved absorption of low-frequency sound can be achieved more effectively by combining open-porous structures with strategically designed air cavities.

### 3.4. Influence of Back Air Cavity

**Figure 7:**
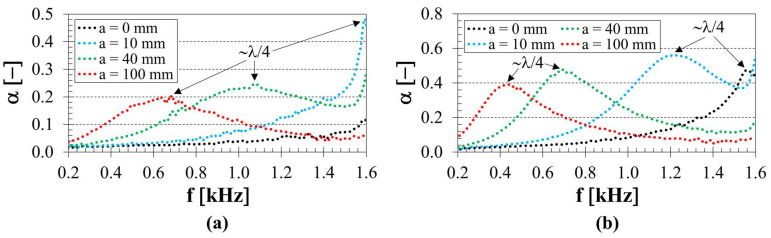
Influence of back air cavity *a* on the frequency dependencies of the sound absorption coefficient; (**a**) number of tubes *T* = 3, strut diameter *d* = 2.5 mm, sample height *h* = 20 mm; (**b**) number of tubes *T* = 5, strut diameter *d* = 2.0 mm, sample height *h* = 30 mm.

The presence of a back air cavity inside the acoustic impedance tube also significantly influences the sound absorption properties of the tested samples. The effect of the back air cavity on the frequency dependencies of the sound absorption coefficient is shown in Figure 7. It is evident that the primary sound absorption maximum (*α_max_*_1_) generally shifts to lower excitation frequencies with increasing back air cavity thickness, resulting in enhanced sound absorption performance of the investigated 3D-printed samples at low frequencies. This phenomenon is associated with the reflection of sound waves from the solid wall of the acoustic impedance tube and with the wavelength *λ*, defined as the ratio of the speed of sound to frequency [53]. At a solid wall, the acoustic pressure is maximal while the air particle velocity is zero. At a quarter-wavelength (*λ*/4) distance, the situation is reversed: the acoustic pressure is zero, and the air particle velocity is maximal. The maximum sound absorption coefficient is achieved when a porous material is placed at a quarter-wavelength distance from the wall, corresponding to back air cavity thicknesses equal to odd multiples of one-quarter wavelength at the following frequencies, as described by Equation (7) [54]:

(7) $$f_{max}=\frac{c\left(2n+1\right)}{4\left(a+h/2\right)}$$

where *c* is the speed of sound, and *n* is an integer (*n* = 0, 1, 2…). Similarly, the minimum sound absorption coefficient is achieved when the back air cavity thickness corresponds to even multiples of one-quarter wavelength at the following frequencies, as given by Equation (8):

(8) $$f_{min}=\frac{c\cdot n}{2\left(a+h/2\right)}$$

The observed shift in the primary absorption peak toward lower frequencies with increasing back air cavity thickness is consistent with the quarter-wavelength resonance principle governing porous absorbers with an air cavity. Similar effects have been widely reported for microperforated panels and porous–cavity configurations, where the back air cavity primarily influences the overall acoustic boundary conditions rather than local dissipative processes [12,27,28,55]. In such configurations, the back air cavity acts as a reactive acoustic element that modifies the effective acoustic impedance of the system, enabling the tuning of resonance conditions without altering the material’s microstructure. This behaviour differs from that represented in Figure 5 and Figure 6, where sound absorption is governed by local geometric parameters and phenomena related to sound propagation within the porous structure.

### 3.5. Influence of Excitation Frequency

Figure 4, Figure 5, Figure 6 and Figure 7 illustrate that the sound absorption properties of the investigated 3D-printed ASA samples were strongly dependent on the excitation frequency of incident acoustic waves. Low sound absorption was generally observed at low excitation frequencies, particularly for samples placed directly on the solid wall inside the acoustic impedance tube, i.e., without a back air cavity (*a* = 0 mm). In these cases, the sound absorption coefficient values were *α* < 0.09 at frequencies below 1000 Hz for all tested samples.

The maximum values of the sound absorption coefficient (*α_max_*) at the corresponding frequency (*f_max_*) obtained for each number of tubes and the corresponding geometric parameters of the tested samples are given in Table 3.

It is evident that the coefficients *α_max_* were obtained at higher frequencies, for greater sample heights and higher strut diameters, and for back air cavity thicknesses ranging from 10 to 40 mm. The highest value of the sound absorption coefficient (*α_max_* = 0.623) was found for the 3D-printed ASA sample fabricated with two concentric tubes, a strut diameter of 3.0 mm, a height of 30 mm, and a back air cavity thickness of 10 mm inside the acoustic impedance tube. In the case of the maximum back air cavity thickness (*a* = 100 mm), the sound absorption peak shifts toward lower frequencies. However, in this frequency range, thin, porous materials exhibit reduced energy dissipation efficiency, resulting in a decrease in the maximum sound absorption coefficient.

The influence of the excitation frequency on the sound absorption performance of the investigated 3D-printed ASA samples was also evaluated based on the average sound absorption coefficient *α_avg_* calculated over the whole measured frequency range. It was found, independently of the number of concentric tubes, that the best average sound absorption properties were obtained for the sample manufactured with a height of 30 mm, a strut diameter of 3.0 mm, and a back air cavity thickness of 10 mm inside the acoustic impedance tube, as described in detail in Section 3.1. The maximum value of the average sound absorption coefficient (*α_avg_* = 0.264) was obtained for this sample, manufactured with five concentric tubes. This value appears relatively low, primarily due to the measurement of the sound absorption coefficient in the low-frequency range of 0.2–1.6 kHz. However, low-frequency sound absorption remains a challenging engineering problem, as long-wavelength acoustic energy is poorly dissipated. Conventional sound-absorbing materials, such as porous media, microperforated panels, and acoustic foams, generally exhibit limited absorption performance in this frequency range [56]. In contrast, these porous sound absorbers usually exhibit good sound absorption properties in the mid- and high-frequency range [57]. Therefore, these novel 3D-printed ASA concentric tubular structures with intermediate lattice inserts can serve as a basis for the development of new material structures that enable more effective sound absorption in the low-frequency range. For example, their geometric parameters, particularly wall thickness and tube profiles, can be optimized, and various infill configurations between the concentric tubes can be investigated.

### 3.6. Comparison with Existing 3D-Printed Sound Absorbers

When comparing the results obtained with previously published 3D-printed sound absorbers, it is clear that the maximum sound absorption coefficient achieved in this study (*α_max_* = 0.623) is lower than that of optimized metamaterial absorbers, which can achieve values of *α* > 0.9 [23,24,25,26,27,28]. However, such high-performance systems typically depend on complex multi-cavity structures, precise micro-scale elements, or multi-component assemblies, which increases manufacturing complexity and reduces robustness in practical applications.

In contrast, the structures proposed in this study are characterized by a single-material, single-process fabrication (FFF), relatively simple and scalable geometry, and good mechanical integrity due to the presence of concentric tubes. Therefore, the main advantage of the present design does not lie in achieving the highest possible peak sound absorption, but rather in providing a balanced combination of manufacturability, structural stability, and tunable acoustic response.

Furthermore, compared to pure lattice structures without outer shells [15,21], the addition of concentric tubes increases airflow resistance and improves average sound absorption. Compared to structures without internal lattice fillers (i.e., empty concentric cavities) [23,28], the lattice inserts cause additional viscous–thermal losses, leading to broader absorption characteristics. This suggests that the proposed hybrid configuration enables a synergistic interaction between global wave propagation and local dissipation mechanisms that is not present in simpler design concepts.

It should be noted that the absolute sound absorption values remain rather moderate compared to state-of-the-art acoustic metamaterials. However, this study focuses more on understanding the structural mechanisms and design trade-offs than on maximizing sound absorption under highly optimized or specific application conditions. In this context, the proposed configuration can serve as a basis for the future development of more efficient sound-absorbing structures, for example, by incorporating alternative or graded internal lattice fillings that can further enhance viscous-thermal dissipation mechanisms.

Overall, the results confirm that the acoustic response of the proposed system can be systematically tuned through geometric design within a hybrid tubular–lattice configuration, highlighting its potential as a tunable design space for porous-resonant sound absorbers.

## 4. Conclusions

This study presented a proof-of-concept investigation of 3D-printed ASA concentric tubular structures with intermediate lattice inserts and analyzed their parametric influence on sound absorption behaviour.

It was found that the sound absorption properties of the investigated samples generally increased with the number of concentric tubes, which was reflected in higher values of the average sound absorption coefficient *α_avg_* over the entire frequency range of 200–1600 Hz. The best average sound absorption properties were obtained for the sample manufactured with a height of 30 mm, a strut diameter of 3.0 mm, and a back air cavity thickness of 10 mm inside the acoustic impedance tube. In this case, the *α_avg_* coefficient decreased from 0.264 (for the sample containing 5 tubes) to 0.192 (for the sample containing 1 tube). Therefore, the *α_avg_* coefficient for the sample containing 5 tubes is approximately 37.5% higher compared to the sample manufactured with 1 tube.

Furthermore, it can be concluded that the sound absorption properties of the studied 3D-printed ASA concentric tubular structures generally increased with increasing strut diameter and sample height. Regarding the back air cavity thickness inside the acoustic impedance tube, a value in the range of 10–40 mm appeared to be the most suitable. The application of a back air cavity with a maximum thickness of 100 mm improved the sound absorption properties of the tested samples at low frequencies. The highest value of the sound absorption coefficient (*α_max_* = 0.623) was found for the sample manufactured with two concentric tubes, a strut diameter of 3.0 mm, a height of 30 mm, and a back air cavity thickness of 10 mm at a frequency of 1548 Hz.

The study primarily presents experimental insight into the relationship between geometry and acoustic response, rather than a new theoretical framework. The observed trends can be explained by changes in well-established mechanisms, such as viscous-thermal dissipation, airflow resistance, and acoustic path length. The results show that, compared to simple porous configurations, the proposed hybrid structures can improve low-frequency sound absorption through geometric tuning of airflow paths and cavity effects. Overall, compared to existing studies, the main contribution of this work lies in demonstrating that relatively simple hybrid structures produced using additive manufacturing can exhibit predictable and tunable acoustic behaviour, representing a promising alternative to more complex metamaterial absorbers in applications where manufacturability and robustness are critical factors.

The investigated 3D-printed concentric tubular structures with intermediate lattice inserts show potential for practical applications in architectural acoustics, transportation systems, and industrial noise control, where lightweight and geometrically optimized sound-absorbing materials are required. Thanks to the design flexibility provided by additive manufacturing, such structures can be tailored to specific frequency ranges and integrated into functional or aesthetic interior elements. In addition, the use of ASA material provides relatively good resistance to environmental factors such as moisture and UV radiation, which may allow its use not only in interiors but also in outdoor acoustic installations.

Future research could focus on optimizing the geometric parameters of concentric tubular structures, in particular the influence of wall thickness and various shape modifications (e.g., elliptical, corrugated or perforated profiles), which can affect the propagation and dissipation of acoustic energy inside the structure. It would also be useful to investigate different types of filling structures between concentric tubes, such as graded or multi-level grids, as well as porous or fibrous fillings that could increase viscous and thermal energy losses. Another possible direction is to study multi-material or functionally graded structures produced by additive manufacturing.

## Figures and Tables

**Table 1:** Physical properties of ASA material [36,37].

**Property**	**Unit**	**Typical Values**
Melt temperature	°C	230
Glass transition temperature	°C	103
Specific Gravity	g/cm^3^	1.08
Shrink rate	%	0.4–0.7
Thermal Conductivity	W·m^−1^·K^−1^	0.1563–0.1685
Ultimate Tensile Strength (UTS)	MPa	32.8 (XY)
Elongation	%	5.9 (XY)
Modulus of elasticity	GPa	2.14 (XY)
Flexural strength at 5% strain	MPa	61.5
Flexural modulus	GPa	1.98
Ultimate Compression Strength	MPa	75.4
Compression modulus	GPa	2.05

**Table 2:** The input parameters for 3D printing jobs.

**Input Parameter Type**	**Value**
Nozzle type	Brass
Nozzle diameter	0.4 mm
Layer height	0.16 mm
First layer height	0.2 mm
Line width	0.42
Seam position	Aligned
Wall generator	Classic
Infill density	100%
First layer speed	50 mm/s
Print speed	80 mm/s
Travel speed	150 mm/s
Supports	No
Brim	No
Shrinkage	99.4%
Print temperature	250 °C
Bed temperature	100 °C
No cooling for	first 3 layers
Part cooling fan speed	10%

**Table 3:** Maximum values of the sound absorption coefficient (*α_max_*) at the corresponding frequency (*f_max_*) obtained for each number of tubes (*T*) together with the corresponding geometric parameters (*d*, *h*, *a*).

*T* (-)	*d* (mm)	*h* (mm)	*a* (mm)	*α_max_* (-)	*f_max_* (Hz)
1	3.0	20	10	0.504	1534
2	3.0	30	10	0.623	1548
3	2.5	20	10	0.485	1590
5	3.0	20	40	0.598	896

## Data Availability

The data that support the findings of this study are openly available in [Zenodo] at https://doi.org/10.5281/zenodo.19367655 [58].
